# *Staphylococcus aureus* and P*seudomonas aeruginosa* infectious keratitis: key bacterial mechanisms that mediate pathogenesis and emerging therapeutics

**DOI:** 10.3389/fcimb.2023.1250257

**Published:** 2023-08-21

**Authors:** Shalini Shah, Rachel A. F. Wozniak

**Affiliations:** Department of Ophthalmology, The University of Rochester School of Medicine and Dentistry, Rochester, NY, United States

**Keywords:** keratitis, bacterial virulence factors, antibiotic drug development, microbial drug resistance, Staphyloccocus aureus, Pseudomonas aeruginosa

## Abstract

Bacterial keratitis (bacterial infection of the cornea) is a major cause of vision loss worldwide. Given the rapid and aggressive nature of the disease, immediate broad-spectrum antibiotics are essential to adequately treat this disease. However, rising antibiotic resistance continues to accelerate, rendering many commonly used therapeutics increasingly ineffective. As such, there is a significant effort to understand the basic pathogenesis of common causative organisms implicated in keratitis in part, to fuel the development of novel therapies to treat this blinding disease. This review explores two common causes of bacterial keratitis, *Staphylococcus aureus* and *Pseudomonas aeruginosa*, with regards to the bacterial mediators of virulence as well as novel therapies on the horizon.

## 1 Introduction

Bacterial keratitis (bacterial infection of the cornea) is a devastating disease responsible for up to 2 million annual cases of blindness globally (Whitcher and Srinivasan, 1997; Ung et al., 2019; Ting et al., 2021c). In the United States, prior studies have reported an incidence of bacterial keratitis of approximately 30/100,000 patients annually (Thylefors et al., 1995), though incidences as high as 700/100,000 have been described in developing nations (Whitcher and Srinivasan, 1997). Overall, ocular trauma remains the greatest risk for bacterial keratitis (Srinivasan et al., 1997; Upadhyay et al., 2001; Bialasiewicz et al., 2006; Ung et al., 2019), however in the United States and other developed nations, other important risk factors include contact lens wear, ocular surface disease, and previous corneal surgery (Erie et al., 1993; Bourcier et al., 2003; Keay et al., 2006).

While a wide variety of bacterial species have been associated with bacterial keratitis, the Gram-positive organism, *Staphylococcus aureus*, and Gram-negative organism, *Pseudomonas aeruginosa*, are among the most commonly identified etiologic agents (Ung et al., 2019; Gurnani and Kaur, 2022). Specifically, studies have reported a prevalence of *S. aureus* keratitis ranging from 8-36% among all cases of bacterial keratitis (Green et al., 2008; Ting et al., 2021c). Of note, the prevalence increases to 24-46% with the inclusion of closely related coagulase-negative *Staphylococcal* species (Tan et al., 2017; Ting et al., 2021c). Similarly, the prevalence of *P. aeruginosa* as causative organism in keratitis, has been reported as high as 10-39% among all cases of infectious keratitis in some centers (Forster, 1998; Varaprasathan et al., 2004; Ting et al., 2021b). Moreover, perhaps due to its propensity to form biofilms on contact lenses and cases, *P. aeruginosa* is a particularly important cause of contact lens-associated keratitis, accounting for nearly 70% of these cases in some series (Galentine et al., 1984; Schein et al., 1989; Cheng et al., 1996; Liesegang, 1997).

Given the clinical severity of bacterial keratitis, initial treatment requires immediate, broad-spectrum topical antibiotics. However, rising antibiotic resistance has rendered many currently available therapeutics ineffective, weakening our ability to adequately treat this blinding disease. For example, among clinical ocular *S. aureus* isolates, resistance to fluoroquinolones, a mainstay of treatment, has been steadily on the rise. Resistance rates as high as 40% have been reported for the third-generation fluoroquinolone, levofloxacin, and 35% for the fourth-generation fluoroquinolone, moxifloxacin (Peng et al., 2018; Thomas et al., 2021). When considering methicillin-resistant *S. aureus* (MRSA), these rates can soar as high as 82% and 73%, respectively (Marangon et al., 2004; Thomas et al., 2019).

Fortunately, among ocular *P. aeruginosa* isolates, circulating resistance has historically remained low in the United States. For example, previous reports of *P. aeruginosa* resistance have ranged from 4% to 17% towards moxifloxacin (Shalchi et al., 2011; Mei et al., 2022). However, in developing countries, resistance has been steadily rising. In India, resistance to fluoroquinolones dramatically rose from 19% to 52% in just over 2 years (Oldenburg et al., 2013; Hernandez-Camarena et al., 2015). Additionally, the appearance of multi-drug resistant *P. aeruginosa* keratitis isolates has been increasingly reported worldwide (Oldenburg et al., 2013; Hernandez-Camarena et al., 2015), including the recent 2023 outbreak of extreme multi-drug resistant *P. aeruginosa* infections associated with contaminated artificial tear solutions that led not only to serious ocular infections but several patient deaths (Shoji et al., 2023).

Given the severity and high prevalence of *S. aureus* and *P. aeruginosa* keratitis, coupled with rapidly rising resistance, there is substantial interest in understanding the underlying bacterial mechanisms driving disease to fuel the discovery of novel therapeutic approaches. As such, this review aims to describe the current literature of known and suspected virulence factors of both *S. aureus* and *P. aeruginosa* that mediate pathogenesis in infectious keratitis (**Table 1**). Additionally, the current state of antimicrobial drug discovery efforts in Ophthalmology is described, highlighting promising new therapies to treat this blinding disease (**Table 2**).

**Table 1:** *S. aureus* and *P. aeruginosa* virulence factors implicated in keratitis.

Virulence Factor	Mechanism of Action	Reference (s)
Staphylococcus aureus		
α-hemolysin toxin	Pore-forming toxin, contributes to corneal ulceration, conjunctival inflammation, corneal edema, iritis, promotes corneal epithelial invasion	(O'Callaghan et al., 1997; Putra et al., 2019)
β-toxin	Sphingomyelinase that promotes lysis of corneal epithelial cell membranes, corneal edema, conjunctival and scleral inflammation	(Dajcs et al., 2002b, O'Callaghan et al., 1997)
γ-toxin	Conjunctival injection and chemosis, corneal edema, corneal infiltrate, hypopyon formation	(Dajcs et al., 2002a; Bierdeman et al., 2017)
PVL	Leukocidin that binds complement pathway proteins, cytotoxic to corneal epithelial cells	(Zaidi et al., 2013)
Fibronectin Binding Protein	Mediates bacterial attachment to corneal cells, promotes corneal epithelial cell invasion	(Jett and Gilmore, 2002; Maurin et al., 2021)
Collagen-adhesin binding protein	Promotes bacterial attachment to the corneal surface	(Rhem et al., 2000)
Staphopain A	Cysteine protease, promotes bacterial binding to fibronectin binding protein	(Hume et al., 2020)
Extracellular adherence protein	Inhibition of host neutrophil proteases, promotes host cellular invasion	(Hume et al., 2020; Kretschmer et al., 2021)
Enterotoxins	*sed, sej, sek, seq, ser, selX*, associated with corneal cell cytotoxicity, increased bacterial burden	(Johnson et al., 2023)
Pseudomonas aeruginosa		
Pseudomonas protease IV	Protease linked to host immune protein degradation, promotes disease severity	(Engel et al., 1998; Traidej et al., 2003; Caballero et al., 2004; Malloy et al., 2005; Thibodeaux et al., 2005)
Exotoxin S, Exotoxin U, Exotoxin T	ExoS associated with invasive infections, ExoU corneal cell cytotoxicity, ExoT mitigates neutrophil-related bacterial killing	(Karthikeyan et al., 2013; Kandasamy et al., 2020, Lomholt et al., 2001, Winstanley et al., 2005; Choy et al., 2008; Stewart et al., 2011; Sun et al., 2012; Borkar et al., 2013)
Small protease (PASP)	Serine protease associated with collagen digestion, corneal ulceration, promotes host immune cell infiltrate	(Tang et al., 2013, Tang et al., 2009)
Elastase (LasB)	Metalloproteinase, correlated with hypopyon and increased corneal ulcer size	(Dart and Seal, 1988; Fleiszig and Evans, 2002, Oka et al., 2015, Thibodeaux et al., 2007)
LPS	Extracellular surface protein, promotes neutrophil infiltration, corneal ulceration, adherence	(Howes et al., 1982a; Zaidi et al., 1996; Schultz et al., 1997)

**Table 2:** Emerging Therapies for Bacterial Keratitis.

Therapeutic	Mechanism of Action	Reference(s)
Pharmaceutical		
Polymyxin B/Trimethoprim + Rifampin	Broad-spectrum antibiotic combination; disrupts bacterial cell membranes (polymyxin B), inhibits DNA synthesis (trimethoprim), inhibits DNA transcription (rifampin)	(Chojnacki et al., 2019a; Chojnacki et al., 2019b; Laskey et al., 2020; Mei et al., 2022)
Cathelicidin/Human beta defensin-2	Hybridized human-derived host defense peptide; Activity against *S. aureus*	(Ting et al., 2021a)
Brilacidin	Synthetic defensin; Activity against *S. aureus* and *Staphylococcus epidermidis*	(Kowalski et al., 2016)
Thymosin beta 4	Human-derived small peptide; mitigates corneal inflammation and enhance *P. aeruginosa* killing	(Sosne and Berger, 2023)
Wedelolactone	Plant-derived protein; attenuates host immune response as adjunctive therapy in *P. aeruginosa* keratitis	(Xu et al., 2021)
Monoclonal Antibody targeting *S. aureus* α-toxin	Decreases corneal ulceration, chemosis, iritis	(Caballero et al., 2015)
Monoclonal Antibody targeting *P. aeruginosa* exotoxins/Type 3 secretion system	Decreases corneal ulceration and opacity	(Hebert et al., 2020)
Non-Pharmaceutical		
PACK-CXL	Stabilizes corneal melting; broad spectrum antimicrobial activity	(Tabibian et al., 2016; Lee et al., 2021; Barac et al., 2022)
Bacteriophage	Reduces disease severity	(Fukuda et al., 2012)
Cold Plasma	Anti-inflammatory properties, promotes wound healing	(Reitberger et al., 2018)

## 2 Staphylococcus aureus

Several key studies have begun to define the set of *S. aureus* virulence factors that are particularly important in promoting virulence in corneal infections. As described below, these include toxins such as α-, β- and γ-toxin, Panton-Valentine leucocidin (PVL), as well as fibronectin binding protein, collagen-adhesion binding protein, Staphopain A, and extracellular adherence protein (Eap), which mediate bacteria-host cell interactions. Additionally, the recent identification of Staphylococcal enterotoxins and their role in keratitis virulence will be discussed (**Table 1**).

The canonical pore-forming toxin, α-hemolysin toxin (*hla*), has been widely implicated in *S. aureus* pathogenesis in diverse physiologic sites as well as shown to promote disease severity in both rabbit and mouse models of keratitis (O'Callaghan et al., 1997). In a study by O'Callaghan et al., α-toxin-deficient strains of *S. aureus* resulted in less conjunctival inflammation and stromal ulceration in rabbit models of keratitis (O'Callaghan et al., 1997). Moreover, purified alpha-toxin applied topically to the rabbit ocular surface led to increased conjunctival inflammation, corneal edema, and iritis, further supporting the role of this virulence factor in keratitis pathogenesis (O'Callaghan et al., 1997). In a complementary study by Putra et al., α-toxin-deficient *S. aureus* strains demonstrated decreased ability to invade corneal epithelial cells in an *in vitro* corneal cell culture and resulted in improved wound healing in a murine model of keratitis (Putra et al., 2019).

β-toxin, a sphingomyelinase that lyses epithelial cell membranes, has also been shown to contribute to keratitis virulence. For example, Dajcs et al. found that in a rabbit keratitis model, infections from *S. aureus* strains deficient in both α- and β-toxins resulted in lower disease severity than strains deficient in α-toxin alone (Dajcs et al., 2002b). The addition of topical purified β-toxin has also been shown to induce mild conjunctival inflammation in rabbit eyes and scleral inflammation in intrastromal injections (O'Callaghan et al., 1997; Dajcs et al., 2002b). Moreover, infections with a β-toxin deficient mutant of *S. aureus* produced less ocular edema in rabbit models than the wild type strains (Dajcs et al., 2002b).

Another notable toxin includes γ-toxin, a pore-forming toxin comprised of two secreted proteins HlgB and either HlgA or HlgC. Using a rabbit model of keratitis, Dajcs et al. demonstrated that while bacterial counts were equivalent in animals infected with *S. aureus* strain Newman and its isogenic *hlg*-deficient mutant, markers of disease severity such as conjunctival injection and chemosis, corneal edema, corneal infiltrate, and hypopyon formation were significantly higher in the presence of γ-toxin (Dajcs et al., 2002a). Additionally, Bierdeman et al. demonstrated that direct injection of γ-toxin into rabbit corneas resulted in significant corneal infiltrates, iritis, fibrin formation in the anterior chamber as well as conjunctival injection (Bierdeman et al., 2017).

Panton-Valentine leucocidin (PVL) is a secreted two component-toxin comprised of the proteins LukF-PV and LukS-PV and is commonly associated with methicillin-resistant *S. aureus* (MRSA) strains. PVL is capable of binding complement pathway proteins and has been shown to have cytotoxic effects to a wide range of host immune cells such as neutrophils, macrophages and natural killer cells (Astley et al., 2019). In a recent study by Zaidi et al., PVL expression in MRSA isolates led to increased bacterial burden and cytotoxicity in both murine and human epithelial cell culture keratitis models (Zaidi et al., 2013). However, interestingly, there is still debate about the clinical importance of PVL as a more recent study on human subjects by Hsaio et al. found similar clinical features, including ulcer location, ulcer size and presence of hypopyon, as well as antibiotic susceptibility between PVL-positive and PVL-deficient strains (Hsiao et al., 2022). It should be noted that there are several other identified homologous protein components such as LukM, LukS-R, LukE, LukF-R and LukD that can combine with each other to form multiple unique toxins. While the roles of these variants have not been explicitly tested in keratitis, it is possible they may also play a role in promoting corneal disease.

Other *S. aureus* virulence factors that have been implicated in keratitis include fibronectin-binding protein, collagen-binding adhesion, Staphopain A, and Eap. Fibronectin-binding protein mediates attachment of bacteria to fibronectin molecules on the surface of corneal epithelial cells which is a precursor for host cellular invasion (Jett and Gilmore, 2002; Maurin et al., 2021). Specifically, Maurin et al. found that fibronectin-binding proteins were the principal driving factor for epithelial invasion, especially in injured epithelium (Maurin et al., 2021). Similarly, collagen-binding adhesion (coa) promotes bacterial attachment to the corneal surface, and in a rabbit model of keratitis, may also promote a more severe suppurative keratitis (Rhem et al., 2000). Additionally, the cysteine protease Staphopain A has been shown to promote *S. aureus* invasion into corneal epithelial cells by increasing fibronectin binding (Hume et al., 2020). Eap has also been found to promote corneal epithelial invasion in murine keratitis models through the inhibition of host neutrophil proteases (Hume et al., 2009; Kretschmer et al., 2021).

Recently, Staphylococcal enterotoxins, a set of secreted toxins well known for their role in *S. aureus*-mediated food poisoning as well as septic shock due to widespread nonspecific host T-cell activation, have been identified as playing a key role in keratitis virulence. For example, in a corneal epithelial cell model, *S. aureus* strains that encode and express one or more of the enterotoxins *sed, sej, sek, seq, ser*, or *selX*, resulted in widespread corneal cell cytotoxicity compared to those infecting strains without enterotoxins. Moreover, in a murine model of keratitis, an infecting strain that expressed enterotoxins Sed, Sej, Sek, Seq and Ser led to a 3-log increase in bacterial burden compared to the isogenic enterotoxin deletion mutant or a strain that naturally lacks enterotoxins (Johnson et al., 2023).

## 3 Pseudomonas aeruginosa

*P. aeruginosa*, similarly to *S. aureus*, has an impressive arsenal of virulence factors that can be leveraged to promote infection in specific physiologic niches. With regards to keratitis, several virulence factors have been described that specifically mediate disease in the cornea. As described below, the serine protease *Pseudomonas* protease IV (PIV), exotoxins *exoS*, *exoU* and *exoT*, *P. aeruginosa* small protease (PASP), Elastases, and extracellular factors such as lipopolysaccharide (LPS) are bacterial determinants that have an established link to keratitis (**Table 1**).

Protease IV has been shown to be a key virulence factor in corneal infections, able to evade the host immune response due to its intrinsic low immunogenicity (Thibodeaux et al., 2005) and promote the degradation of key host immune proteins such as complement, fibrinogen, plasminogen and immunoglobulin (Engel et al., 1998; Malloy et al., 2005). While PIV does not appear to impact the bacterial burden in a rabbit model of keratitis, disease severity as assessed by slit lamp exams was significantly higher in strains expressing PIV compared to an PIV-deficient mutant (Caballero et al., 2004). Moreover, plasmid-based expression of PIV in an otherwise PIV-negative *Pseudomonas putida* strain led to significant increase in corneal tissue damage and ocular inflammation (Traidej et al., 2003).

Two other significant *P. aeruginosa* keratitis virulence factors are exotoxin S (*exoS*), which is implicated in invasive infections, and exotoxin U (*exoU*), which is associated with acute host cytotoxicity (Fleiszig et al., 1997; Karthikeyan et al., 2013; Kandasamy et al., 2020). Both proteins are secreted by the *P. aeruginosa* Type III secretion system and generally speaking, isolates either encode either *exoS* or *exoU*. While some studies have found no difference in the prevalence of these strains in keratitis isolates (Lomholt et al., 2001), others have found a propensity for *exoU*-positive strains in cases of keratitis, particularly in patients with contact lens-associated disease (Winstanley et al., 2005; Choy et al., 2008; Stewart et al., 2011). Moreover, the large-scale Steroids for Corneal Ulcers Trial (SCUT) found that those patients infected with *exoU*-positive strains had larger corneal infiltrates and presented with poorer visual acuity compared to patients infected with *exoS*-positive strains (Borkar et al., 2013). Two other effector proteins secreted by the *P. aeruginosa* Type III secretion system include ExoT, which is a closely related protein to ExoS, and ExoY, an adenylate cyclase. While ExoY appears to have limited influence in keratitis virulence, ExoT has been shown to promote *P. aeruginosa* disease severity as well as mitigate neutrophil-mediated bacterial killing in a murine model of keratitis (Sun et al., 2012). Of note, the Type III secretion system itself is a critical requirement for corneal disease, as strains that do not assemble this secretion system at all result in attenuated clinical disease severity in mice (Sun et al., 2012).

*P. aeruginosa* small protease (PASP) is a serine protease that causes collagen digestion, thereby promoting corneal epithelial and stromal ulceration (Marquart et al., 2005; Tang et al., 2013). Moreover, purified PASP injection into the cornea resulted in a significant polymorphonucleated cell stromal infiltrate (Tang et al., 2009). In both a corneal scratch and intrastromal injection rabbit model of keratitis, while the overall bacterial load remained unchanged, PASP-deficient mutants resulted in reduced disease severity as measured by slit-lamp exam scores, and infected eyes demonstrated less inflammation and tissue damage as measured by histology (Tang et al., 2013). Elastase (LasB) is another example of a metalloproteinase that can disrupt corneal extracellular matrix proteins and, in some models, has been shown to promote *P. aeruginosa* keratitis virulence (Dart and Seal, 1988; Fleiszig and Evans, 2002; Thibodeaux et al., 2007). The presence of Elastase was also significantly correlated with hypopyon formation and larger corneal ulcer size in human patients with *P. aeruginosa* keratitis (Oka et al, 2015). However, the exact role of LasB, as well as the related elastase, LasA, in corneal virulence is not entirely clear, as several studies have demonstrated that isogenic deletion mutants of *lasB* and *lasA* did not result in disease attenuation in a mouse model of keratitis (White et al., 2001; Hobden, 2002).

Extracellular surface factors, including pili and polysaccharide capsule component LPS, are also implicated in promoting *P. aeruginosa* keratitis disease severity through potent host immune stimulation (Fukuda et al., 2017). For example, injection of LPS into the rabbit corneal stroma led to rapid infiltration of neutrophils as well as corneal ulceration (Howes et al., 1982b; Schultz et al., 1997). Additionally, LPS may also have a role in promoting *P. aeruginosa* adherence to corneal epithelial cells as well as subsequent bacterial internalization (Zaidi, 1996).

## 4 Emerging therapies

As rising antibiotic resistance threatens our current therapeutic arsenal, there is an urgent need for novel antimicrobial therapeutics. While pharmaceutical companies have largely abandoned antimicrobial drug development in favor of more long-term, lucrative therapeutics, academic centers continue to innovate, seeking to leverage the expanding knowledge of bacterial pathogenesis to develop new treatment strategies. As described below, there are several novel therapeutics on the horizon to treat bacterial keratitis including novel broad-spectrum antibiotic drug combinations, host defense peptides, monoclonal antibodies, and non-pharmaceutical approaches such as corneal cross linking, bacteriophage and plasma therapies.

One strategy in the field of antimicrobial drug discovery is to repurpose existing therapeutics in new combinations to elicit improved antimicrobial activity. To that end, the combination of polymyxin B-trimethoprim (PT) + rifampin has recently been shown to display broad-spectrum, synergistic antimicrobial activity, with rapid *in vitro* bactericidal activity and anti-biofilm activity, the latter of which may have particular importance given the propensity of bacteria to establish biofilms on contact lenses (Chojnacki et al., 2019a; Chojnacki et al., 2019b). The potent activity of this combination has also been shown to effectively eradicate large sets of *S. aureus* and *P. aeruginosa* ocular clinical isolates from around the world, including those strains displaying multi-drug resistance in *in vitro* studies, demonstrating the ability to PT + rifampin to overcome relevant circulating isolates (Laskey et al., 2020; Mei et al., 2022). Importantly, PT + rifampin has been shown to eradicate *in vivo S. aureus* and *P. aeruginosa* keratitis infections in a murine model. For example, following infection with a clinical keratitis isolate of *S. aureus* with known fluoroquinolone-resistance, topical treatment with PT + rifampin four times daily for 72 hours led to complete eradication of disease in 7 out of 10 animals, compared to a less than 2-log reduction in bacterial burden following treatment with topical moxifloxacin, a current gold standard of treatment (Chojnacki et al., 2019b). Given the resistance to fluoroquinolones is on the rise, this data suggests that PT + rifampin may be a viable alternative for the treatment of keratitis.

An additional approach in drug discovery is to exploit naturally produced antimicrobial host immune compounds. For example, human-derived host defense peptides (HDPs), are a class of proteins produced by a variety of immune cells and are well known for their broad-spectrum antimicrobial activity via direct bacterial cell lysis. While naturally occurring HDPs have established limitations such as weak stability and host toxicity, several groups have sought to modify these compounds to improve compatibility and efficacy. For example, a synthetic, hybridized HDP comprised of cathelicidin and human beta defensin-2 components has been shown to have potent antimicrobial activity towards *S. aureus* in *in vitro* studies, a low propensity to drive resistance, and a modest reduction in bacterial burden in a *S. aureus* model of keratitis (Ting et al., 2021a).

A similar study has explored the efficacy of brilacidin, a synthetic mimetic of defensins, compounds naturally produced by innate immune cells that exhibits immunomodulatory activities as well as antibacterial effects via a variety of mechanism such as depolarizing bacterial cell membranes, neutralizing toxins, and inhibition of cell wall synthesis (Kowalski et al., 2016). Kowalski et al. demonstrated *in vitro* efficacy of brilacidin towards both *S. aureus* and *Staphylococcus epidermidis*, and, in a rabbit model of MRSA keratitis, brilacidin displayed equivalent efficacy compared to vancomycin with no evidence of toxicity. Of note, in this model, the efficacy of brilacidin required an ulcerated epithelium, suggesting there may be poor penetrance of this compound with an intact ocular surface (Kowalski et al., 2016).

Another proposed small peptide with a potential role in treating infectious keratitis is thymosin beta 4, a small, ubiquitously found natural protein that has been shown to reduce inflammation in the cornea and enhance bacterial killing in *P. aeruginosa*-associated keratitis (Sosne and Berger, 2023). Currently, thymosin beta 4 is being actively investigated in the treatment of dry eye disease due to its anti-inflammatory and pro-wound healing properties. However, as an adjunctive therapy with ciprofloxacin, thymosin beta 4 improved inflammatory infiltrates and enhanced bacterial killing in a *P. aeruginosa* murine keratitis model compared to a ciprofloxacin-only treated group. Similarly, studies have explored the use of the plant-derived compound wedelolactone as an adjunctive therapy to antibiotics to mediate inflammatory injury in *P. aeruginosa* keratitis. Wedelolactone exhibits diverse antioxidant and anti-inflammatory properties and is thought to mitigate non-canonical programmed cell death. As an adjunctive therapy with ciprofloxacin, wedelolactone resulted in reduced disease severity and attenuated immune response in a rat model of *P. aeruginosa* keratitis (Xu et al., 2021).

The use of monoclonal antibodies has also been explored as a potential treatment of infectious keratitis. For example, a human monoclonal antibody Fab fragment to *S. aureus* α-toxin has been evaluated in a rabbit model of *S. aureus* keratitis, demonstrating that this approach can decrease corneal ulceration, chemosis and iritis (Caballero et al., 2015). Similarly, a monoclonal antibody that targets *Pseudomonas* exotoxins and type III secretion proteins was shown to be non-toxic to the ocular surface in mice as well as mitigated signs of infection (Hebert et al., 2020).

Other novel therapeutic approaches include the use of non-pharmaceutical approaches such as photoactivated chromophore corneal collagen cross-linking (PACK-CXL), bacteriophage therapy, and plasma therapy. Corneal cross-linking is well-known for its application in slowing the progression of corneal ectatic disorders. However, it has also been appreciated that the free-radicals generated from the combination of ultraviolet-A light and the photosensitizing agent, riboflavin, may have antimicrobial activity as well. Over the past 10 years, PACK-CXL has been used to treat advanced corneal melts, including those with infectious origins, both as a standalone treatment but also in conjunction with traditional antimicrobial therapies with moderate success (Papaioannou et al., 2016; Tabibian et al., 2016; Lee et al., 2021; Barac et al., 2022). While this technology is currently used in cases that have failed conventional treatment, with optimization of both the light source and photosensitizing agent, there may be broader applications in the future.

Bacteriophages are viruses that naturally infect and kill specific bacterial species, making their use in the treatment of human disease appealing. While a major limitation of bacteriophage therapy is the narrow spectrum of activity of individual phages, there have been recent studies demonstrating efficacy in select cases of bacterial keratitis. Fukuda et al. have shown that a single-dose administration of a bacteriophage targeting the *P. aeruginosa* strain PA33 was sufficient to reduce disease severity and the host immune response (Fukuda et al., 2012). Additionally, there is also a recent case report of a human patient with recalcitrant *S. aureus* infection successfully treated with phage therapy (Fadlallah et al., 2015).

Finally, cold plasma is an ionized gas that already has established applications in medicine given its anti-inflammatory and wound healing effects. Recently this technology has been tested for its antibacterial activity as well as for safety on corneal limbal epithelial cells, suggesting there may be some promise in this novel technology for the treatment of keratitis (Reitberger et al., 2018).

## 5 Conclusion

Infectious keratitis is a blinding disease and current therapeutic options are failing due to the relentless increase in antibiotic resistance. To combat circulating and emerging drug-resistant strains, new antimicrobial therapies are desperately needed. This review highlights both the basic pathogenesis of two common causes of keratitis, *S. aureus* and *P. aeruginosa* in corneal infections as well as the leading edge of new therapies in the pipeline. An increasing knowledge of the bacterial drivers of infections can fuel future discoveries to both directly target the pathogen and mitigate the damaging host response.

## Author contributions

RW edited the manuscript and SS conducted literature review. Both RW and SS contributed to the manuscript writing. All authors contributed to the article and approved the submitted version.
